# Autoradiography Imaging in Targeted Alpha Therapy with Timepix Detector

**DOI:** 10.1155/2015/612580

**Published:** 2015-01-22

**Authors:** Ruqaya AL Darwish, Alexander Hugo Staudacher, Eva Bezak, Michael Paul Brown

**Affiliations:** ^1^Department of Medical Physics, Royal Adelaide Hospital, Adelaide, Australia; ^2^School of Chemistry and Physics, University of Adelaide, Adelaide, Australia; ^3^Translational Oncology Laboratory, Centre for Cancer Biology, SA Pathology and University of South Australia, Adelaide, Australia; ^4^School of Medicine, University of Adelaide, Australia; ^5^Cancer Clinical Trials Unit, Royal Adelaide Hospital, Adelaide, Australia

## Abstract

There is a lack of data related to activity uptake and particle track distribution in targeted alpha therapy. These data are required to estimate the absorbed dose on a cellular level as alpha particles have a limited range and traverse only a few cells. Tracking of individual alpha particles is possible using the Timepix semiconductor radiation detector. We investigated the feasibility of imaging alpha particle emissions in tumour sections from mice treated with Thorium-227 (using APOMAB), with and without prior chemotherapy and Timepix detector. Additionally, the sensitivity of the Timepix detector to monitor variations in tumour uptake based on the necrotic tissue volume was also studied. Compartmental analysis model was used, based on the obtained imaging data, to assess the Th-227 uptake. Results show that alpha particle, photon, electron, and muon tracks were detected and resolved by Timepix detector. The current study demonstrated that individual alpha particle emissions, resulting from targeted alpha therapy, can be visualised and quantified using Timepix detector. Furthermore, the variations in the uptake based on the tumour necrotic volume have been observed with four times higher uptake for tumours pretreated with chemotherapy than for those without chemotherapy.

## 1. Introduction

The relationship between the characteristics of a particular radiation type and its impact on irradiated biological tissues and cells plays an important role in estimating the efficacy and applicability of targeted radiotherapies [1]. In a specific case of targeted alpha therapy (TAT), a tumour-specific antibody or protein is radiolabelled with an alpha-emitting radionuclide, termed a radioimmunoconjugate [2, 3]. This radioimmunoconjugate attaches preferentially to tumour-specific antigens that can be expressed on a tumour cell membrane and release high-linear energy transfer (LET) alpha particles with kinetic energy of a few MeV. The traversal of these short-ranged alpha particles through target and neighbouring tumour cells results in localised radiation damage and ultimately cell death [2, 4]. TAT can, however, also result in so-called cross fire irradiation in which antigen-negative nontumour cells in close vicinity of the radioimmunoconjugate are also irradiated and damaged. The magnitude of this radiation damage (to tumour or healthy cells) strongly depends on the tumour-specific uptake of the particular radioimmunoconjugate.

To date, there is a lack of methods that can quantify alpha particle emissions in biological systems. One of the few devices presently available (known as the “*α*-camera”) combines autoradiography with a scintillation detector and optical registration using a charge-coupled device. In the study of Bäck and Jacobsson, the distribution of At-211 labelled antibody and antibody fragments in human tumour, mouse kidney, and whole-body sections were examined [6]. The *α*-camera had a resolution of 35 ± 11 *µ*m, and the quantitative analysis proved that the pixel intensity has a linear relationship with the activity of the imaged tissue. The results demonstrated the ability of the *α*-camera to be employed in small-scale dosimetry for TAT as it was able to provide quantitative data on a microscopic scale [6]. Furthermore, the *α*-camera was also used to examine the accumulation of an At-211 radioimmunoconjugate administered to mice with ovarian cancer micrometastases. The activity level and the number of tumour cell clusters were determined by imaging one section with the *α*-camera and by staining a consecutive section with hematoxylin and eosin [7]. The study demonstrated that the radioimmunoconjugate had high uptake and retention at the tumour surface and that dose estimates to micrometastases could be calculated using the *α*-camera.

**Figure 1:**
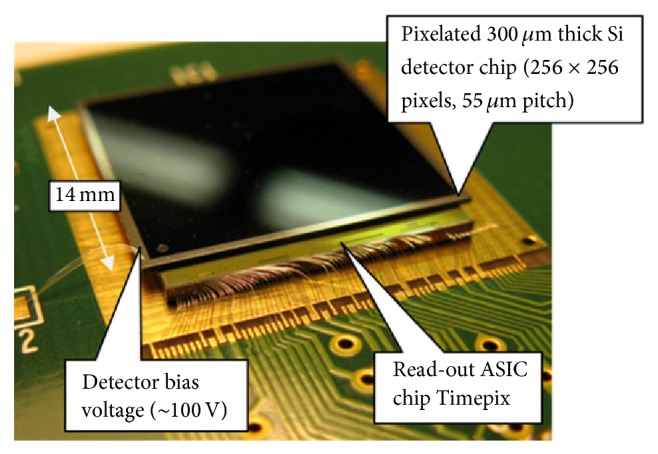
Timepix detector structure, courtesy of [5].

Timepix is a new prototype radiation detector which takes advantage of recent developments of the complementary metal-oxide-semiconductor (CMOS) technology for constructing integrated circuits. Timepix consists of a silicon semiconductor layer, divided into an array of pixels, which is bumped-bonded to an electronics integrated layer (Figure 1). Each pixel is connected to an individual charge-sensitive preamplifier, a discriminator and a counter [8, 9], and a 4-bit digital-to-analogue converter (DAC) to adjust the voltage threshold (4-bit DAC for threshold adjustment) [10].

Timepix is a sophisticated microdosimeter that can be used for a wide range of experiments with photons, electrons, and other charged particles and has applications in fields such as space physics, nuclear physics, radiotherapy physics, imaging, and radiation protection. One of its main advantages is that it can measure energy deposition directly and in real time [11]. For example, in the work of Esposito et al., Timepix was used to trace *β*-particles froma C-14 sample [12, 13]. C-14 was deposited on a low-density paper foil, evaporated before packing in 10 *μ*m thick Mylar, and being read by the detector [12]. The image was analysed in terms of clusters of hit pixels, which gave an indication of the interaction position of the *β* particle with the detector. The result showed that Timepix was highly sensitive with a minimum detectable activity of 0.0077 Bq and with a spatial resolution of 76.9 *μ*m at full width at half maximum (FWHM) [13].

In this paper, we present the first results for using Timepix to visualise TAT* ex vivo *in mouse tumour sections. As mentioned above, the *α*-emitting radioimmunoconjugate binds to its cancer-specific antigen. The emitted *α* particles deposit their kinetic energy inside a target cell, as well as in surrounding cells, potentially resulting in cell death. In the current work, the DAB4 murine monoclonal antibody (trademarked as APOMAB), which binds to necrotic tumour cells [14, 15], was used and was radiolabelled with Thorium-227 as described in [4]. Since there is a spatial correlation inside a tumour between the necrotic and the hypoxic regions [4], we hypothesised that targeting or binding of DAB4 to necrotic tumour regions would result in nearby hypoxic tumour cells receiving cross dose and hence undergoing radiation-induced death. Hypoxic tumour cells are generally resistant to low LET radiation like that of X-rays, which is typically employed in clinical radiation therapy. Furthermore, by increasing the number of dead tumour cell targets, for example, after chemotherapy, the tumour uptake of the radioimmunoconjugate would be increased, consequently increasing the tumour dose [16]. In order to confirm this hypothesis, qualitative and quantitative detection and analysis of the radioimmunoconjugate uptake and its distribution within the tumour volume are required, using suitable microdosimetric detection techniques. In this study, we used the Timepix microdosimeter to detect radiation emissions from tumour sections of mice treated with Thorium-227 APOMAB to image and quantify alpha particle emissions at a micrometre level.

## 2. Materials and Methods

### 2.1. Th-227

**Figure 2:**
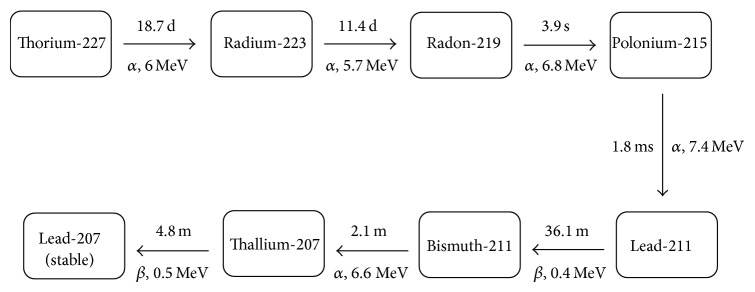
The decay chain of Th-227, half-lives, and mean energies of emitted particles.

The radionuclide Thorium-227 is an alpha emitter produced from actinium-227 with a half-life of 18.7 days, making it, attractive for use in therapeutic applications [17, 18]. Alpha particles have a short range of only a few cell diameters in tissue (<100 *μ*m [18]). The higher LET of alpha particles results in greater biological effectiveness compared to other radiation types such as X-rays or electrons [19]. Along with alpha particle emissions, the Th-227 decay chain also results in the release of *β*-particles and a low percentage of X- and gamma rays prior to reaching a stable isotope (Lead-207). The Th-227 decay chain and the mean energies of major particles produced in this decay chain are presented in Figure 2. Th-227 used in the current work was purchased through the National Isotope Development Centre, Department of Energy, USA.

### 2.2. Preparation of Autoradiography Sample

#### 2.2.1. Monoclonal Antibody Production, Conjugation, and Radiolabelling with ^227^Th

The La-specific murine monoclonal antibody DAB4 (APOMAB) [20] in conjugation buffer (0.1 M sodium bicarbonate, 0.1 M monosodium phosphate, and pH 8.5) was mixed with 50-fold molar excess of the bifunctional chelator p-SCN-Bn-DOTA (Macrocyclics, USA) and incubated at room temperature for four hours with rotation. After buffer-transfer to 0.5 M sodium acetate buffer (pH 5), the ratio of DOTA : mAb was determined as previously described [21] and was approximately 4 : 1.

^227^Th was purified as previously described [18] immediately prior to radiolabelling. DAB4-p-SCN-DOTA was incubated overnight with purified Th-227 using an Eppendorf Thermomixer (37°C, 550 rpm constant). Samples were washed three times and buffer-transferred to phosphate-buffered saline (pH 7.4). The activity of the radioimmunoconjugate was determined using a Germanium detector and MCDWIN version 3.08 software (FAST ComTec, Germany), with the main gamma peaks of 236 and 256 keV (17.6% and 9.5% abundance, resp.) used to quantify Th-227 activity and of 269 and 154 keV peaks (13.9% and 5.7% abundance, resp.) used to quantify Radium-223. The specific activity of radioimmunoconjugates was 400 kBq/mg, with <1% Ra-223 and <1% unbound Th-227 present, as determined by instant thin-layer chromatography.

#### 2.2.2. LL2 Tumour Model and Treatment of Tumour-Bearing Mice

All animal experiments were approved by the SA Pathology Animal Ethics Committee, Adelaide, and conducted following institutional ethical guidelines. Six- to eight-week-old female C57Bl/6 mice were injected subcutaneously in the right flank with 10^6^ LL2 cells (this cell line is derived from transplantable murine Lewis lung carcinoma). Tumour size was measured using electronic callipers, and tumour volume was determined using the following calculation: tumour  volume = (*a* ^2^ × *b*)/2, where* a *is the shortest diameter and* b* is the longest diameter of the tumour. Treatment commenced when tumours reached 45–60 mm^3^. To generate more necrotic tumour cells for Th-227-DAB4 binding, some mice also received chemotherapy prior to injection with Th-227-DAB4. These were treated intravenously with 50 mg/kg gemcitabine (Hospira, Australia) on days 1 and 2 and 2.5 mg/kg cisplatin (Hospira) on day 1. 18 kBq of ^227^Th-DAB4 was administered on day 3. Mice were euthanised 2 days after administration of Th-227 labelled antibody via cervical dislocation, and tumours were collected and fixed in 10% neutral-buffered formalin. Tumours were paraffin embedded, and 5 *µ*m sections were cut for imaging.

### 2.3. Timepix Radiation Detector

The Timepix radiation detector, used in the current work, was purchased from Amsterdam Scientific Instruments (ASI), the Netherlands. The device consists of a silicon chip, 1.408 × 1.408 cm^2^ in size containing 256 × 256 pixels, with each pixel having an area of 55 × 55 *µ*m^2^ and 300 *µ*m depth.

The chip can collect positive or negative charges [9], and the range of particle energies to be detected can be selected for a uniform performance using adjustable thresholds. The device can be operated in one of three main modes to either count single particles (Medipix mode), measure the arrival time of events/particles (Timepix mode), or measure the energy deposited in each pixel for events between the thresholds (time over threshold (TOT) mode) [10]. This offers the possibility of using the detector for a wide range of applications for photon and particle detection and energy spectrometry in addition to imaging and tomography.

The ASI Timepix detector is combined with the beam data acquisition software, SoPhy (Software for Physics), developed by the provider. The SoPhy software allows one to control Timepix acquisition modes such as selection of the operation mode, adjustment of suitable energy thresholds, cluster sizes, and other acquisition settings using multiwindows. The recorded data and frames/images can be exported to and processed using other programs such as Matlab or ImageJ.

**Figure 3:**
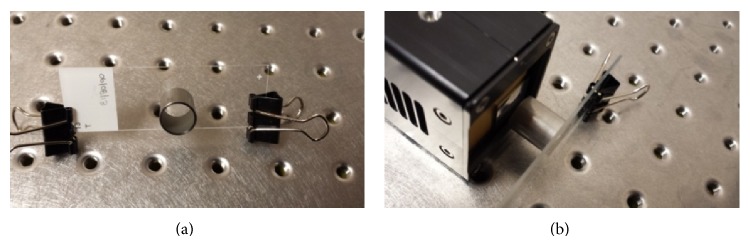
Image of the experimental setup. (a) Tumour section with a 2 cm diameter collimator mounted on top of a tumour section. (b) A tumour section in front of the uncovered Timepix detector.

In the current work, before any measurements and following manufacturer recommended procedure, pixel equalisation was performed to ensure a uniform response of all pixels. Subsequently, tumour sections mounted on glass microscope slides were placed 2 cm away from the detector with the front face of the detector uncovered to allow the emitted alpha particles to penetrate the Timepix silicon chip (Figure 3). A simple steel collimator with 1 cm radius and 2 cm in length was manufactured in-house and positioned around the tumour section using epoxy glue. Bias voltage of 5 V was applied to Timepix. The TOT mode was selected along with alpha particle filter. This acquisition filter allows identification of individual alpha particles detected (amongst other detected particles) as well as determination of the centre pixel in a charge cluster produced in the Timepix silicon chip by a traversing alpha particle. The total acquisition time was approximately 14 hours with 0.01 seconds per frame. Individual frames as well as the total integrated image can be evaluated. In the current work, the detected number of alpha particle hits from individual samples was evaluated and compared after correction for decay and normalisation for tumour section size and acquisition time.

### 2.4. Compartmental Analysis

**Figure 4:**
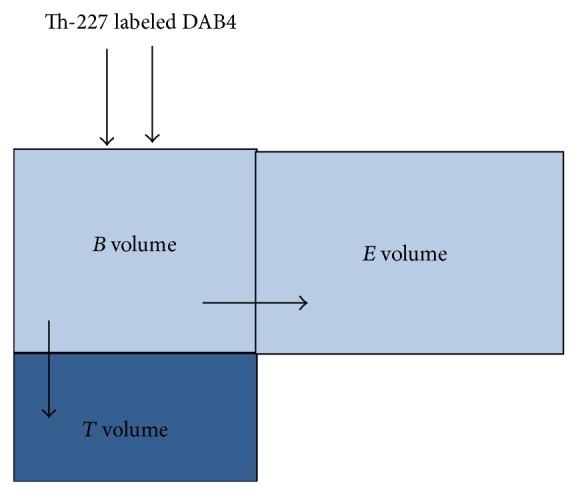
Compartmental model for the autoradiography using Th-227 labelled DAB4.

The compartmental model [22] was used in the current work to calculate and compare the radioimmunoconjugate uptake in TAT-treated tumours. It is assumed that there are three main compartment volumes: the blood volume (*B*), tumour volume (*T*), and a free volume (*E*), where the radioactive source will escape from the blood. It is assumed that, after injection, the Th-227-DAB4 will distribute throughout all volumes. The blood and the escape volumes are open volumes where the radiolabelled immunoconjugate can escape from. The tumour volume, however, is a closed volume where the taken-up Th-227-DAB4 concentration will remain steady as shown in Figure 4.

For simplicity, it is assumed in the current work that, at time zero, the initial activity concentration of the isotope in blood, *C*_*B*_*o*__, is equal to the administered activity, *A*_*o*_; that is, all of the injected activity has been taken up by blood. Subsequent changes of the Th-227-DAB4 concentration in the blood volume, *dC*_*B*_/*dt*, are due to isotope uptake by the tumour and due to excretion/depletion through escape volume. This can be expressed as [2, 22]

(1) $$\begin{array}{c} \frac{dC_{B}}{dt}=-k_{T}C_{B_{o}}-k_{E}C_{B_{o}},\\ C_{B}\left(t\right)=A_{o}e^{-\left(k_{T}+k_{E}\right)t}, \end{array}$$

where *k*_*T*_ and *k*_*E*_ are the so-called transfer (uptake) coefficients for tumour and for the escape volumes.

Variations in Th-227-DAB4 concentration in tumour volume, *dC*_*T*_/*dt*, are due to Th-227 uptake from the blood volume and due to its depletion through radioactive decay of Th-227. This can be then written as

(2) dCTdt=kTCBot−λCT,dCTdt=kTAoe−(kT+kE)t−λCT,

where *λ* is the decay probability constant for Th-227.

Assuming, for simplicity, that the tumour volume transfer coefficient, *k*_*T*_, is much higher than escape volume transfer coefficient, *k*_*E*_, the previous equation becomes

(3) $$\begin{array}{c} \frac{dC_{T}}{dt}=k_{T}A_{o}e^{-k_{T}t}-\lambda C_{T},\\ C_{T}\left(t\right)=\frac{k_{T}}{\lambda -k_{T}}A_{o}\left(e^{-k_{T}t}-e^{-\lambda t}\right). \end{array}$$

In our case, for tumour sections treated with only Th-227-DAB4, the concentration of the radioactive material at time *t* can be expressed as

(4) $$\begin{array}{c} C_{T,\text{nochemo}}\left(t\right)=\frac{k_{T,\text{nochemo}}}{\lambda -k_{T,\text{nochemo}}}A_{o}\left(e^{-k_{T,\text{nochemo}}t}-e^{-\lambda t}\right). \end{array}$$

Similarly, for tumour sections treated with chemotherapy prior to Th-227-DAB4 administration, the concentration of Th-227 at time *t* can be expressed as

(5) $$\begin{array}{c} C_{T,\text{chemo}}\left(t\right)=\frac{k_{T,\text{chemo}}}{\lambda -k_{T,\text{chemo}}}A_{o}\left(e^{-K_{T,\text{chemo}}t}-e^{-\lambda t}\right). \end{array}$$

The ratio of radioisotope concentrations between the radiolabelled tumour samples with and without chemotherapy is

(6) CT,chemo(t)CT,nochemo(t)=e−kT,chemot−e−λte−kT,nochemot−e−λt=e−kT,chemot1−e−λ−kT,chemoe−kT,nochemot1−e−λ−kT,nochemo.

Considering that the decay probability coefficient *λ* is much smaller compared to the transfer coefficient *k*_*T*_, the above ratio can be reduced to

(7) $$\begin{array}{c} \frac{C_{T,\text{chemo}}\left(t\right)}{C_{T,\text{nochemo}}\left(t\right)}=\frac{e^{-k_{T,\text{chemo}}t}-1}{e^{-k_{T,\text{nochemo}}t}-1}. \end{array}$$

Once the mouse has been euthanised and/or all of the initial activity has been taken up by the tumour and escape volumes (e.g., at time *t*_max⁡_), the amount of radioisotope in the tumour volume,* C*_*T*_, will only vary as a result of radioactive decay:

(8) $$\begin{array}{c} \frac{dC_{T}}{dt}=-\lambda C_{T}\left(t_{\max}\right). \end{array}$$

As both tumour sample types (treated and not treated with chemotherapy) will decay with the same decay probability, the number of recorded alpha emissions will be directly proportional to the amount of radioisotope taken up by the tumour prior to a mouse being euthanatised. As a result, from the measurement point of view, the number of alpha hits detected by Timepix detector is proportional to isotope concentrations, *C*_*T*,chemo_ and *C*_*T*,nochemo_, during measurements. As a result, from the number of recorded alpha hits measured at different time intervals, the transfer coefficients for the two scenarios could be determined.

## 3. Results and Discussion

**Figure 5:**
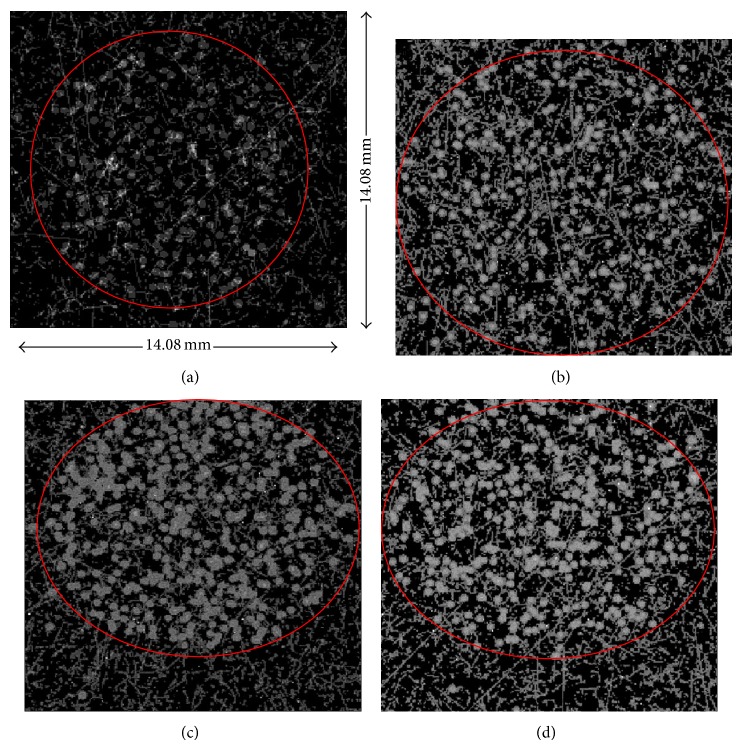
(a) and (b): images of tumour sections from mice treated with ^227^Th-DAB4 alone. (c) and (d): images of two tumour sections from mice treated with chemotherapy followed by ^227^Th-DAB4. The red circle indicates the approximate tumour section boundaries.

Figure 5 shows acquired integrated images of sections from four tumours: two tumours from mice treated with ^227^Th-DAB4 alone ((a), (b)) and two tumours from mice treated with chemotherapy followed by ^227^Th-DAB4 ((c), (d)). Even though the collimator was positioned around the tumour section, the collimator was not touching the detector, leaving a small (~2 mm) air gap. As a result, particles emitted at smaller angles, compared to a trajectory perpendicular to the detector, can still reach the detector. These are observed as hits outside the red circle in Figure 5.

**Figure 6:**
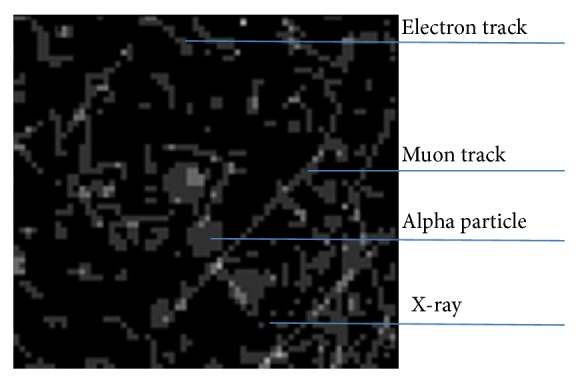
Timepix responses to electron, Muon, X-/gamma ray, and alpha particle.

High LET alpha particles release their kinetic energy in more than one pixel as they pass in the semiconductor detector, forming a charge cluster. The small dots recorded in the image represent X- and gamma rays. An electron passage will result in a wavy and short track, and a muon track will be detected as a straight, long line (Figure 6).

An automated method using ImageJ was used to determine the number of alpha particle hits. These were also counted manually for comparison. The differences between the two methods varied between 4 and 20 hits. Using the automated method, the number of hits varied between 93 and 251, for the sections that did not receive chemotherapy, and between 445 and 582, for the sections that were treated with chemotherapy before administering Th-227-DAB4.

**Figure 7:**
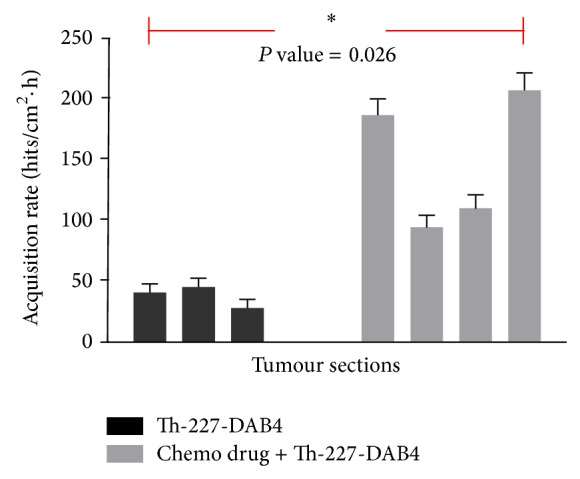
Measured alpha particle hits per unit tumour area per 1 hour for two groups of tumour sections: 4 sections with and 3 sections without application of chemotherapy prior to administration of Th-227 radioimmunoconjugate.

As shown in Figure 7, the recorded alpha particle hits for each sample were corrected for radioactive decay (based on the time elapsed between the Th-227 administration and Timepix measurement), the area of each sample, and total acquisition time, resulting in the alpha particle acquisition rate between 27.6 and 44.8 hits/cm^2^·hour for tumour sections of mice not previously given chemotherapy and between 94.0 and 206.8 hits/cm^2^·hour for sections that were treated with chemotherapy followed by ^227^Th-DAB4.

Corrected alpha particle acquisition rates were statistically analysed using one-way ANOVA for their significance, using GraphPad Prism software, yielding* P* value of 0.026 (<0.05, two-tailed), confirming that the observed differences between the two sample groups are significant.

**Figure 8:**
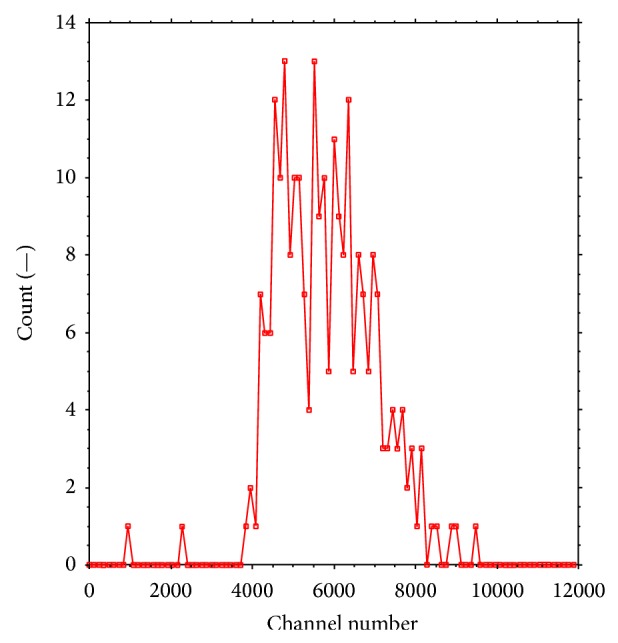
The detected alpha particle spectrum as emitted from Th-227-DAB4 from a single tumour section.

Energy spectra of alpha particles detected from decay of Th-227 and its daughters were also acquired and an example is presented in Figure 8 where the lowest and the highest energies can be determined. The energy varies between 4 and 7.4 MeV, corresponding to the expected energy range of alpha particles produced by Th-227 decay chain.

Using the compartmental analysis, corresponding transfer coefficients for the two samples can be determined when repeating the measurements for at least one sample type at different times. The data can be plotted using (4) or (5) and the transfer coefficient can be estimated. The ratio in detected alpha particles between the two sample types was found to be approximately 4. If one of the transfer coefficients is known from repeated measurements, the second one can be determined from (7).

## 4. Conclusion

Tumour sections were imaged in the current work to characterise the pattern of uptake and distribution of Th-227-DAB4. High resolution autoradiographs of radiolabelled tumour sections were acquired, showing alpha particles, *β* particles, electrons, and X-ray tracks. The Timepix measurements also showed an increased uptake of Th-227–DAB4 following chemotherapy due to an increase in necrotic tissue volume. The experiment showed that the Timepix detector can be used effectively for autoradiography in TAT to provide high-resolution images. Development of a fine collimator can improve the definition of the tumour boundaries and identify the geometrical origin of detected alpha particles within the sample. It was also shown that, in principle, the data acquired by Timepix can be used for compartmental analysis to quantify the uptake of radioimmunoconjugate in targeted alpha therapy.
